# Translation and Validation of the Medication Understanding and Use Self‐Efficacy Scale Among Patients With Type 2 Diabetes in Taiwan

**DOI:** 10.1111/hex.70222

**Published:** 2025-03-15

**Authors:** Yen‐Ming Huang, Yu‐Meng Yang, Tzu Wang, Yunn‐Fang Ho, Hsun‐Yu Chan

**Affiliations:** ^1^ Graduate Institute of Clinical Pharmacy, College of Medicine, National Taiwan University Taipei City Taiwan; ^2^ School of Pharmacy, College of Medicine, National Taiwan University Taipei City Taiwan; ^3^ Department of Pharmacy National Taiwan University Hospital Taipei City Taiwan; ^4^ Department of Industrial Education National Taiwan Normal University Taipei City Taiwan

**Keywords:** diabetes, medication, self‐efficacy, translation, validation

## Abstract

**Objective:**

The study aimed to translate and assess the validity and reliability of the Traditional Chinese version of the Medication Understanding and Use Self‐Efficacy Scale (MUSE‐TC) among patients with type 2 diabetes (T2D) in Taiwan.

**Methods:**

The original 8‐item MUSE was translated into Traditional Chinese using a forward and backward translation method. The translations were reviewed by four experts in pharmacy practice and educational psychology. The validity and reliability of the MUSE‐TC were assessed in a cross‐sectional study among adults with T2D who were taking diabetes medications. Participants were recruited from five community pharmacies in Taiwan between June 2023 and May 2024. Internal consistency of the MUSE‐TC was measured using McDonald's omega (ω), while construct validity was evaluated through confirmatory factor analysis. Criterion validity was established by examining the relationship between self‐efficacy and medication adherence.

**Results:**

A total of 274 patients participated in the study. Confirmatory factor analysis identified a two‐factor structure for the 8‐item MUSE‐TC, consisting of the “taking medication” and “learning about medication” domains. All items loaded onto their intended factors, with factor loadings ranging from 0.433 to 0.511. The scale demonstrated excellent internal consistency, with McDonald's ω values of 0.914 for the “taking medication” domain and 0.906 for the “learning about medication” domain. These robust psychometric properties were further supported by criterion validity, as self‐efficacy was shown to be associated with medication adherence. Specifically, the “taking medication” domain was positively correlated with fewer barriers to medication‐taking (*r* = 0.382, *p* < 0.001), suggesting that individuals with higher self‐efficacy tend to adhere to their prescribed medication regimen.

**Conclusions:**

This study presents the MUSE‐TC with psychometrically sound properties that will enable healthcare professionals to prospectively assess self‐efficacy in medication use and evaluate the impact of self‐efficacy on a variety of health outcomes across patients with different chronic diseases.

**Patient or Public Contribution:**

Patients, pharmacists, pharmacy staff, and academic experts worked together on the translation and validation of this self‐efficacy scale for medication adherence among individuals with chronic illnesses. Community engagement played a key role in translating the scale, recruiting participants, collecting data, conducting analysis, and interpreting the findings. These collaborative efforts ensure the study relevance and applicability to patient care in routine healthcare settings.

## 1 Introduction

Optimal medication adherence is essential for achieving effective disease management and positive patient health outcomes [1]. Despite the availability of numerous effective pharmacological therapies for managing chronic conditions, adherence rates remain around 50% [2], posing a major obstacle to therapeutic success [3]. Medication non‐adherence often reflects a mismatch between patient and medical priorities or may result from complex medication regimens that overwhelm an individual's capacity [4]. This issue is widely associated with poor health outcomes and unnecessary healthcare costs [5]. Medication‐taking behaviour is influenced by a complex interplay of biological, psychological, and social factors [6]. Modifiable risk factors for poor adherence include negative treatment attitudes, prior non‐adherence, substance abuse, short illness duration, inadequate discharge planning, poor after‐care environments, and weak therapeutic alliances [7, 8]. Among these, psychosocial factors offer practical solutions that can be addressed in clinical settings. A meta‐analysis revealed that self‐efficacy has a stronger positive correlation with medication adherence compared to other cognitive factors, such as illness or treatment beliefs [9]. Consequently, self‐efficacy is recognised as a salient factor that can be integrated in clinical interventions to enhance medication adherence [10].

Self‐efficacy, a core concept in Social Cognitive Theory, refers to an individual's belief in their ability to organise and carry out the actions needed to achieve specific goals [11]. Unlike a personality trait, self‐efficacy is behaviour‐specific and can be learned and strengthened over time [12]. This makes it particularly useful in patient education and healthcare intervention, where the focus is not only on increasing knowledge and coping skills but also on empowering individuals for better self‐management [7]. In recent years, self‐efficacy theory has been applied to understand medication adherence behaviours [13]. Numerous healthcare studies have highlighted its role in both initiating and maintaining medication regimens [14, 15, 16]. Research generally indicates that individuals with chronic conditions who have high levels of self‐efficacy and positive attitudes toward self‐management tend to adhere to behaviours like taking and refilling medications [4]. Clinical trials further support this by showing that self‐efficacy enhances medication adherence and contributes to improved clinical outcomes, such as better glycemic control [17, 18] and reduced HIV viral load [19]. Given its influence on the effort and persistence patients exert in overcoming challenges to adherence, self‐efficacy is a key factor in designing interventions to improve medication adherence [20]. As a result, assessing self‐efficacy is a useful strategy for developing tailored interventions to address suboptimal medication adherence [21].

Research highlights the predictive value of self‐efficacy in medication adherence among patients with chronic diseases [22, 23], leading to the development of various instruments to assess self‐efficacy in medication management [4]. These include the Long‐Term Medication Behaviour Self‐Efficacy Scale (LTMBSES) [24, 25], Medication Adherence Self‐Efficacy Scale (MASES) [26], Self‐Efficacy and Outcome Expectations for Osteoporosis Medication Adherence Measures (SEOMA and OEOMA) [27], Self‐efficacy for Appropriate Medication Use Scale (SEAMS) [28], HIV Treatment Adherence Self‐Efficacy Scale (HIV‐ASES) [29], Medication Understanding and Use Self‐Efficacy Scale (MUSE) [30], and Diabetes Medication Self‐efficacy Scale (DMSS) [31]. Despite the availability of these tools, most are disease‐specific (e.g., LTMBSES, MASES, SEOMA, OEOMA, HIV‐ASES, and DMSS) [25, 26, 27, 29, 31] or lengthy [26], limiting their applicability across different chronic conditions [25]. Although self‐efficacy is crucial for understanding and predicting health behaviours [32], it remains underassessed in Taiwan, as no valid and reliable instrument exists in Traditional Chinese to evaluate this psychological factor related to medication use. Without an accurate tool to measure self‐efficacy in medication management, enhancing adherence through self‐efficacy‐based strategies in clinical practice is challenging [33].

The MUSE is a brief, valid, and reliable questionnaire designed for both clinical practice and research to assess patients’ understanding and use of prescription medications [30]. Unlike existing disease‐ or context‐specific measures, the MUSE takes a more general approach to evaluating self‐efficacy in medication use. In addition to assessing self‐efficacy, the MUSE highlights the importance of patients’ understanding of their medications [4]. Originally developed in English by Cameron et al. to reflect respondents’ understanding and confidence in taking prescription medications [30], the MUSE has also been translated and validated in a Malaysian version for patients with diabetes, demonstrating validity and reliability comparable to the original version [33]. This study aimed to translate the MUSE into Traditional Chinese (MUSE‐TC) and evaluate its validity and reliability among patients with Type 2 diabetes (T2D) in community settings.

## 2 Methods

### 2.1 Study Design and Setting

In this cross‐sectional study, the MUSE was first translated into Traditional Chinese by the research team after receiving permission from the original author [30]. The translated MUSE‐TC was then administered to eligible participants through face‐to‐face interviews at five community pharmacies in northern and western Taiwan between June 2023 and May 2024. The study protocol was approved by the Research Ethics Committee at National Taiwan University Hospital (202303022RIND). Written informed consent was obtained from all participants before their involvement in the study.

### 2.2 Instrument

The MUSE is an 8‐item scale designed to measure two key constructs of self‐efficacy in understanding and using prescription medications, including both taking medication (4 items) and learning about medication (4 items) [30]. It was originally developed from two subscales of the Communication and Attitudinal Self‐Efficacy Scale [34], with additional items added to capture respondents’ understanding of and confidence in managing their prescription medications. This self‐administered scale uses a four‐point Likert scale (1 = *strongly disagree*, 4 = *strongly agree*) to assess an individual's self‐efficacy in medication use and generate a total score ranging from 8 to 32. Higher scores indicate greater confidence in learning about and taking medication. The MUSE has demonstrated good internal reliability, with Cronbach's *α* values of 0.77 for the subscale on taking medication and 0.68 for learning about medication [30]. Together, the two subscales account for 55% of the total variance in understanding medication instructions and offer a comprehensive assessment of self‐efficacy across various literacy levels [30].

### 2.3 Instrument Translation

The transcultural adaptation of the MUSE for its use with the Taiwanese population followed the Principles of Good Practice for the Translation and Cultural Adaptation Process for Patient‐Reported Outcomes Measures [35]. The translation process involved both forward and backward translation. Two native Chinese‐speaking researchers proficient in English translated the scale from English into Traditional Chinese. The researcher, along with bilingual experts involved in the translation, held discussions to ensure that the translated scale accurately conveyed the original meanings and aligned with the cultural context in Taiwan. A bilingual pharmacist then back‐translated the initial translation into English. The research team compared the original and back‐translated English versions, consulted the translators and advisors, resolved any discrepancies, and produced a final consensus version.

To ensure content validity, four experts in pharmacy practice and educational psychology evaluated the translation and cultural adaptation of each item during the pre‐expert review phase. The draft scale was then tested through cognitive interviews with 10 individuals with T2D, selected via convenience sampling, to assess item clarity and the time required to complete the scale. A think‐aloud method was used to gauge the comprehensibility and ease of understanding for each item in the Traditional Chinese MUSE version [36]. As no major revisions were needed, the final version, MUSE‐TC, was administered to the study participants (Appendix A).

### 2.4 Instrument Validation

#### 2.4.1 Participant Recruitment

We used convenience sampling to recruit eligible individuals aged 18 or older who were diagnosed with T2D, prescribed at least one oral medication for diabetes management, and able to read Traditional Chinese. The National Health Insurance MediCloud System was utilised to verify participants’ diagnosis (i.e., International Classification of Diseases, Tenth Revision, Clinical Modification diagnosis code of E11) and their diabetes medications. Patients were excluded if they lacked active electronic health records (EHRs), could not understand Traditional Chinese, or had cognitive impairments, such as dementia or Alzheimer's disease.

At the five participating community pharmacies, pharmacists and staff assisted with participant screening and referred eligible individuals to speak with the trained researcher. The researcher, experienced in working with this patient population and trained in structured interviewing techniques, took eligible patients to a private area, described study details, distributed information sheets, and allowed 5–10 min for review. Interested patients were invited to sign the informed consent form and were given a copy of the unsigned informed consent form for their records. Following consent, the researcher administered the survey and collected participants’ clinical data via the MediCloud System. The paper‐and‐pencil survey consisted of 23 items and typically took 3–5 min to complete. Participants were compensated with US$7 upon survey completion.

There is no consensus on the rigid sample size for confirmatory factor analysis (CFA). However, prior recommendations suggest a minimum of 10 cases per variable, with at least 200 subjects considered sufficient for evaluating the psychometric properties of instruments that measure social constructs [36]. Given that the MUSE‐TC includes 8 items, this recommendation indicated a minimum sample size of 200 subjects for conducting CFA.

#### 2.4.2 Measures

In addition to administering the MUSE‐TC, we collected data on participants& self‐reported medication adherence as well as their sociodemographic and clinical characteristics. The Traditional Chinese version of the Adherence to Refills and Medications Scale (ChARMS‐T) was used to evaluate participants’ self‐reported adherence to prescribed diabetes medications. Specifically, we focused on the 8‐item medication‐taking subscale of the ChARMS‐T, which evaluates barriers to medication intake that are closely related to individual beliefs [37]. This subscale has shown strong internal consistency, with a McDonald's omega (ω) of 0.841, and demonstrated robust construct validity in individuals with T2D [37]. Each item is scored on a 4‐point Likert scale (1 = *none of the time*, 2 = *some of the time*, 3 = *most of the time*, 4 = *all of the time*), resulting in a total score ranging from 8 to 32. For better interpretation, the responses were reverse‐scored so that higher scores indicate better medication adherence and fewer barriers to taking medication.

Sociodemographic data included self‐reported age, gender, educational attainment, and annual household income. Clinical characteristics comprised the number of prescribed diabetes medications and use of injectable diabetes treatments. Verification of participants’ diabetes medications was completed by cross‐referencing their medication histories in EHRs.

#### 2.4.3 Statistical Analysis

Descriptive statistics were used to summarise the characteristics of the study participants. For continuous variables, the mean and standard deviation (SD) were provided, while categorical variables were presented as counts and percentages. The reliability of the scale was assessed through item‐total correlations [38], while internal consistency was evaluated using McDonald's ω [39]. An item‐total correlation exceeding 0.30 was considered satisfactory [38], and ω values of 0.7 or higher indicated acceptable or strong internal consistency [39].

Both construct validity and criterion validity were employed to validate the MUSE‐TC. Construct validity was assessed using CFA, as prior literature has clearly delineated the items associated with specific dimensions of the MUSE [30]. Confirmatory factor analysis was conducted to determine how well the data fit a model based on existing theoretical or empirical work [40]. Initially, we examined the correlation matrix of all 8 items and calculated their inter‐item correlations to determine the strength of associations between them. The goodness‐of‐fit for the factor structure was evaluated using the comparative fit index (CFI), Tucker‐Lewis index (TLI), root mean square error of approximation (RMSEA), and standardised root mean square residual (SRMR). Values exceeding 0.95 for CFI and TLI, along with an RMSEA value below 0.05, indicate an acceptable model fit. Additionally, RMSEA and SRMR values below 0.05 suggest a “good fit” [40].

Once the final scale was established, we assessed criterion validity by comparing composite self‐efficacy scores with ChARMS‐T scores. Previous studies indicate that self‐efficacy is linked to patients’ medication‐taking behaviours [41]. Thus, we hypothesised that higher self‐efficacy regarding medication‐taking would positively correlate with improved medication adherence, as measured by the ChARMS‐T. Pearson correlation coefficients were applied to evaluate this hypothesis.

Since complete data for the MUSE‐TC scale were available in this sample, no imputation methods were necessary. Descriptive statistics and the correlation matrix were calculated using the Statistical Package for the Social Sciences version 28, while McDonald's ω and CFA were performed using JASP version 0.19.1. The significance level was set at a two‐sided *p* < 0.05.

## 3 Results

### 3.1 Participants' Characteristics

A total of 315 individuals were invited to participate, and 274 (87.0%) enroled and completed the survey. Of these, the majority were female (*n* = 142, 51.8%), with ages ranging from 28 to 94 years and an average age of 67.64 years (SD = 10.61). Educational backgrounds varied, with 27.0% holding a high school diploma, followed by 20.1% with a bachelor's degree, and 19.7% having completed junior college. Additionally, 50.7% (*n* = 139) reported an annual household income of NT$660,000 (approximately US$22,000) or more. Participants were prescribed an average of 1.96 ± 0.98 medications for diabetes management, with 21 (7.7%) using both oral hypoglycemic agents and injectable medications for diabetes control (Table 1).

**Table 1:** Demographic and clinical characteristics of the participants (*n* = 274).

Variables	*n* (%)	Mean (SD)
Age (years)		67.64 (10.61)
Gender		
Female	142 (51.8)	
Male	132 (48.2)	
Educational attainment		
Elementary school	38 (13.9)	
Junior high school	30 (10.9)	
High school	74 (27.0)	
Junior college	54 (19.7)	
Bachelor's degree or a 4‐year college degree	55 (20.1)	
Master's degree or above	23 (8.4)	
Annual household income		
Less than 350,000	76 (27.7)	
350,001–659,999	59 (21.5)	
660,000–959,999	58 (21.2)	
960,000–1,309,999	53 (19.3)	
1,310,000–2,209,999	18 (6.6)	
Above 2,210,000	10 (3.6)	
Number of diabetes medication		1.96 (0.98)
Use of injectable diabetes medication	21 (7.7)	
Duration of diabetes diagnosed (years)		11.77 (9.19)
Self‐report diabetes medication adherence (score = 8–32)		29.47 (2.63)
Medication understanding and use self‐efficacy (score = 8–32)		25.17 (3.59)
Taking medication (score = 4–16)		12.59 (2.00)
Learning about medication (score = 4–16)		12.58 (1.89)

### 3.2 Validity Evaluation of Factor Structure of the MUSE‐TC

The CFA confirmed the factor structure of the MUSE‐TC, indicating that a two‐factor model provided a good fit to the data (*χ* ^2^ = 26.858, *df* = 19, *p* = 0.108, CFI = 0.954, TLI = 0.932, SRMR = 0.046, RMSEA = 0.039). Factor loadings ranged from 0.433 to 0.511 (Table 2). The two identified factors, taking medication and learning about medication, had a correlation coefficient of 0.700 (*p* < 0.001) (Table 3). For criterion validity, the hypothesised relationship for validating the MUSE‐TC component was confirmed. The taking medication domain positively correlated with fewer barriers to medication‐taking (*r* = 0.382, *p* < 0.001) (Table 3).

**Table 2:** Confirmatory factor analysis for the Medication Understanding and Use Self‐Efficacy Scale in Traditional Chinese version.

	Questions	Factor 1 (taking medication)	Factor 2 (learning about medication)
1.	It is easy for me to take my medicine on time.	0.498	
2.	It is easy to remember to take all my medicines.	0.439	
3.	It is easy for me to set a schedule to take my medicines each day.	0.511	
4.	It is easy for me to take my medicines every day.	0.433	
5.	It is easy for me to ask my pharmacist questions about my medicine.		0.474
6.	It is easy for me to understand my pharmacist's instructions for my medicine.		0.448
7.	It is easy for me to understand instructions on medicine bottles.		0.453
8.	It is easy for me to get all the information I need about my medicine.		0.451

**Table 3:** Bivariate correlations of the study variables.

	(1)	(2)	(3)	(4)	(5)	(6)	(7)	(8)	(9)	(10)
Age (1)	1.000									
Gender (2)^a^	−0.146*	1.000								
Educational attainment (3)	−0.294***	0.424***	1.000							
Annual household income (4)	−0.261***	0.211***	0.405***	1.000						
Use of injectable diabetes medication (5)^b^	0.032	−0.003	0.068	−0.065	1.000					
Number of diabetes medication (6)	0.006	0.118	0.082	0.043	0.387***	1.000				
Diabetes duration (7)	0.323***	0.033	−0.055	−0.065	0.275***	0.502***	1.000			
Score of medication‐taking (8)	0.221***	−0.066	−0.187**	−0.064	−0.189**	−0.136*	0.036	1.000		
Self‐efficacy score‐Taking medication (9)	0.034	0.065	0.081	0.054	0.042	−0.040	0.036	0.382***	1.000	
Self‐efficacy score‐Learning about medication (10)	−0.122*	0.069	0.182**	0.134*	0.015	−0.041	−0.025	0.160**	0.700***	1.000

^a^ Female was used as the comparison group.

^b^ Did not use injectable diabetes medication was used as the comparison group.

* *p* < 0.05,

** *p* < 0.01,

*** *p* < 0.001

### 3.3 Reliability Assessment

The item‐total correlation coefficients for the MUSE‐TC ranged from 0.715 to 0.802, with McDonald's ω values ranging between 0.857 and 0.902 if any of the 8 items were removed. Overall, the MUSE‐TC showed excellent internal consistency, with a McDonald's ω of 0.914 for the “taking medication” domain and 0.906 for the “learning about medication” domain. The mean scores and standard deviations for the 8 MUSE‐TC items are presented in Table 4.

**Table 4:** Reliability analysis the Medication Understanding and Use Self‐Efficacy Scale in Traditional Chinese version.

	Questions	Mean (SD)	Item‐total correlation	McDonald's ω if item removed
Taking medication: McDonald's ω = 0.914				
1.	It is easy for me to take my medicine on time.	3.12 (0.58)	0.762	0.888
2.	It is easy to remember to take all my medicines.	3.13 (0.56)	0.736	0.902
3.	It is easy for me to set a schedule to take my medicines each day.	3.14 (0.58)	0.802	0.866
4.	It is easy for me to take my medicines every day.	3.19 (0.54)	0.751	0.897
Learning about medication: McDonald's ω = 0.906				
5.	It is easy for me to ask my pharmacist questions about my medicine.	3.15 (0.52)	0.757	0.880
6.	It is easy for me to understand my pharmacist's instructions for my medicine.	3.17 (0.49)	0.795	0.857
7.	It is easy for me to understand instructions on medicine bottles.	3.15 (0.55)	0.739	0.886
8.	It is easy for me to get all the information I need about my medicine.	3.11 (0.58)	0.715	0.896

### 3.4 Distribution of the MUSE‐TC and Relations With Other Variables

The mean score for the medication‐taking domain was 12.59 (range: 8–16; SD = 2.00), while the mean score for the domain of learning about medication was 12.58 (range: 7–16; SD = 1.89) (Table 1). The majority of participants (*n* = 266, 97.1%) demonstrated strong self‐efficacy in both taking and learning about their medication. Higher self‐efficacy in learning about medication was associated with higher educational attainment (*r*_s_ = 0.182, *p* = 0.003), greater household income (*r*_s_ = 0.134, *p* = 0.026), younger age (*r* = 0.122, *p* = 0.043), and fewer barriers to medication adherence (*r* = 0.160, *p* = 0.008) (Table 3).

## 4 Discussion

The validation study of the MUSE‐TC indicates that this scale exhibits satisfactory psychometric properties for assessing self‐efficacy in medication understanding and use among patients with T2D in community settings. The psychometric properties of the MUSE‐TC appear to be strong and comparable to those of the original English version [30]. The construct validation conducted through CFA revealed two components of the MUSE‐TC, namely the taking medication domain and the learning about medication domain. This finding mirrors the pattern seen in the original English version [30] as well as in another validation study involving patients with diabetes in Malaysia [33]. This supports the robustness of the original MUSE and reinforces the strength of its initial validation. Furthermore, McDonald's ω coefficients indicated excellent internal consistency reliability for both domains of the translated MUSE‐TC. Therefore, the MUSE‐TC retains the psychometric qualities of the original version and offers a valid and reliable assessment of self‐efficacy in medication use among patients with T2D in Taiwan.

As expected, medication adherence was significantly associated with self‐efficacy in both taking and learning about medication. These findings align with previous studies showing that higher self‐efficacy is positively associated with better medication adherence, with patients reporting stronger self‐efficacy adhering more consistently to their medication regimen [42, 43]. Since the MUSE‐TC was significantly related to medication adherence, the scale could help providers identify individuals who are at higher risk of non‐adherence. Healthcare professionals can use this tool to screen patients who lack confidence in specific aspects of taking or understanding their medications and offer tailored strategies to address these challenges. Successful self‐management of chronic diseases depends largely on behaviour, and individuals with higher self‐efficacy are more likely to implement necessary lifestyle changes and maintain healthy behaviours [44]. Therefore, the MUSE‐TC serves as a useful tool for assessing and enhancing self‐efficacy, which is crucial for improving medication adherence and achieving long‐term control of chronic conditions.

Unlike generic self‐efficacy measures, the MUSE‐TC captures broader domains to assess self‐efficacy in medication use compared to existing disease‐ or context‐specific measures, while also acknowledging the importance of patients’ understanding of their medications [30]. Many available self‐efficacy scales related to medication use focus primarily on a patient's belief in their ability to take medication as suggested [4]. In contrast, some scales have been specifically designed for particular diseases or conditions [25, 26, 27, 29, 31]. While this focus on specific barriers or conditions is commendable because self‐efficacy is behaviour‐specific [12], not all health behaviours are necessarily related to individualised conditions. Therefore, the availability of a more general scale can be beneficial for both clinical and research purposes. Healthcare professionals may find it impractical to have multiple self‐efficacy measures tailored to each patient's specific condition. Additionally, since many patients are prescribed medications for multiple comorbidities, asking them to respond to numerous condition‐specific scales could be cumbersome and confusing. Time constraints and busy workflows further complicate this issue. Therefore, the brevity of the 8‐item MUSE‐TC makes it highly valuable for clinicians and researchers, as it allows for the assessment of patients’ self‐efficacy in learning about and taking their medications across various disease states.

The study findings highlight the importance of addressing self‐efficacy to improve medication adherence, particularly among older adults and individuals with lower educational attainment and household income. Self‐efficacy is a critical and modifiable factor in improving medication adherence in healthcare settings. Understanding the contributors to low self‐efficacy is essential for developing behaviour change interventions [26]. These findings echo existing literature, which shows that lower education levels [45], older age [46], and lower household income [47] are linked to reduced self‐efficacy in medication use across various conditions. Patients with lower education and income levels may have lower health literacy and pay less attention to their health, further contributing to diminished self‐efficacy in adhering to medications [48]. Moreover, the financial burden of long‐term medications for chronic conditions may be more significant for lower‐income individuals, reducing their access to treatments [49]. Therefore, it is essential to recognise the role of self‐efficacy in populations with lower income or education levels to inform targeted interventions. Additionally, older adults may experience cognitive decline and lower health literacy, which can reduce their confidence in managing medications as prescribed by healthcare professionals [50]. Enhancing self‐efficacy in older patients with multiple comorbidities and low medication literacy is critical for improving adherence. Incorporating social cognitive and behavioural therapies in psychological treatment could help boost self‐efficacy and promote better medication adherence in this population [50]. In summary, the translated MUSE‐TC is well‐suited for assessing patients’ self‐efficacy regarding their prescribed medications within the cultural context of Taiwan.

### 4.1 Strengths and Limitations

Following Cameron et al.'s [30] recommendation, this study used a proximal outcome (i.e., adherence to prescribed medications) to evaluate the validity of the MUSE‐TC. This approach advances and strengthens this area of research by providing detailed psychometric insights that link self‐efficacy to actual behaviour rather than relying on proximal outcomes, such as comprehension of medication instructions.

Interpreting the findings of this study requires consideration of its methodological limitations. Self‐report questionnaires are prone to inherent biases, such as social desirability and recall bias. However, these biases may have been mitigated to some extent through the rigorous development and validation processes of the questionnaire. The study sample consisted solely of patients with T2D, so it is unclear if the results would differ for individuals with other symptomatic conditions (e.g., arthritis). Further research involving individuals with other diseases is necessary to enhance the generalisability of the findings. While the scale used in this study demonstrated excellent internal consistency, it was not subjected to test‐retest analysis to evaluate its stability over time. This underscores the importance of conducting such reliability testing in future research. Moreover, the discrepancy in time frames between self‐efficacy and adherence behaviours may limit the broader applicability of the findings. Specifically, self‐efficacy was measured based on participants' current confidence, while adherence measures reflected pills missed in the past. As a result, the data do not support causal inferences. Future research employing the MUSE‐TC in longitudinal studies and with adherence measures will greatly contribute to this field. Lastly, non‐response bias cannot be ruled out, as not all patients invited to participate consented to complete the questionnaire, potentially excluding certain groups from the analysis.

### 4.2 Practice Implications

Improving self‐efficacy can potentially enhance medication adherence, a multifaceted behaviour shaped by physical, psychological, cognitive, social, and economic factors [51]. The MUSE‐TC provides researchers with a tool to assess self‐efficacy related to medication use in patients with chronic conditions. It can help identify patients at risk for non‐adherence or evaluate the effectiveness of interventions aimed at boosting self‐efficacy. Incorporating the MUSE‐TC into educational interventions may strengthen these programs by not only increasing knowledge but also improving self‐efficacy for medication adherence and promoting proper medication use [52]. By identifying areas of low self‐efficacy before an intervention, the scale can be used both before and after to examine the intervention impact on self‐efficacy.

The current findings provide direction for future research on the role of self‐efficacy in key outcomes. On one hand, prospective studies could clarify the causal relationship between MUSE‐TC scores and medication adherence, healthcare utilisation, and clinical outcomes over time. For example, it remains unclear whether more advanced disease status causes or results from lower self‐efficacy for medication adherence. A valuable area of exploration would be tracking MUSE‐TC scores as patients begin therapy and are monitored over time. On the other hand, intervention studies could examine whether and how self‐efficacy factors are modifiable and whether improvements in MUSE‐TC scores lead to better medication adherence and improved clinical outcomes over time. Additionally, investigating how MUSE‐TC factors relate to patterns of healthcare utilisation could offer insights into the economic impact of self‐efficacy and the potential cost‐effectiveness of interventions targeting self‐efficacy in medication adherence [29].

## 5 Conclusions

The MUSE‐TC shows adequate psychometric properties for assessing self‐efficacy in medication adherence among patients with T2D in community pharmacies in Taiwan. A clear association between the MUSE‐TC and medication adherence has been established. The MUSE‐TC serves as a valuable and brief tool for clinicians and researchers to evaluate self‐efficacy in both learning about medications and adhering to prescribed regimens. Ongoing refinement and further testing of the MUSE‐TC scale are recommended, especially in broader patient populations and over longer follow‐up periods.

## Author Contributions

**Yen‐Ming Huang:** conceptualisation, methodology, software, data curation, supervision, resources, formal analysis, validation, investigation, funding acquisition, writing – original draft, writing – review and editing, project administration. **Yu‐Meng Yang:** conceptualisation, methodology, investigation, data curation, project administration, validation, writing – review and editing. **Tzu Wang:** investigation, validation, methodology, writing – review and editing, project administration. **Yunn‐Fang Ho:** conceptualisation, methodology, supervision, writing – review and editing. **Hsun‐Yu Chan:** conceptualisation, methodology, supervision, software, resources, funding acquisition, writing – review and editing.

## Disclosure

Parts of this work were presented at the 14th Pharmaceutical Care Network Europe Working Conference in Innsbruck, Austria, on 5 February 2025.

## Ethics Statement

This study was approved by the Research Ethics Committee of National Taiwan University Hospital (202303022RIND). All procedures adhered to the principles of the Declaration of Helsinki.

## Consent

Before participation, all individuals provided written informed consent, which also covered consent for the publication of anonymized data.

## Conflicts of Interest

The authors declare no conflicts of interest.

## Data Availability

The study materials and detailed analyses are available from the corresponding author upon reasonable request.

## Appendix A

See Table A1

**Table A1:** The Traditional Chinese version of the Medication Understanding and Use Self‐Efficacy Scale.

		非 常 不 同 意	不 同 意	同 意	非 常 同 意
1.	對我而言, 準時吃藥是容易的。	○	○	○	○
2.	對我而言, 記得吃所有的藥是容易的。	○	○	○	○
3.	對我而言, 規畫每天吃藥的時間表是容易的。	○	○	○	○
4.	對我而言, 每天吃藥是容易的。	○	○	○	○
5.	對我而言, 向藥師詢問有關我的用藥問題是容易的。	○	○	○	○
6.	對我而言, 瞭解藥師提供給我的用藥指導是容易的。	○	○	○	○
7.	對我而言, 瞭解藥品包裝上的用藥指示是容易的。	○	○	○	○
8.	對我而言, 獲得我需要的藥品相關資訊是容易的。	○	○	○	○
